# Eye tracker as an implied social presence: awareness of being eye-tracked induces social-norm-based looking behaviour

**DOI:** 10.16910/jemr.12.2.5

**Published:** 2019-08-05

**Authors:** Hoo Keat Wong, Ian D. Stephen

**Affiliations:** University of Nottingham Malaysia, Semenyih, Malaysia; Macquarie University, Sydney, Australia

**Keywords:** Eye movement, eye tracking, implied social presence, looking behaviour, region of interest, social attention

## Abstract

Human behaviour is not only influenced by the physical presence of others, but also implied social presence. This study examines the impact of awareness of being eye-tracked on eye movement behaviour in a laboratory setting. During a classic yes/no face recognition task, participants were made to believe that their eye movements were recorded (or not recorded) by eye trackers. Their looking patterns with and without the awareness of being eye-tracked were compared while perceiving social (faces, faces-and-bodies) and non-social (inanimate objects) video stimuli. Area-of-interest (AOI) analysis revealed that misinformed participants (who were not aware that their eye movements were being recorded) looked more at the body (chest and waist) compared to informed participants (who believed they were being eye-tracked), whereas informed participants fixated longer on the mouth and shorter on the eyes of female models than misinformed participants did. These findings highlight the potential impact of an awareness of being eye tracked on one’s eye movement pattern when perceiving a social stimulus. We conclude that even within laboratory settings an eye tracker may function as an implied social presence that leads individuals to modify their eye movement behaviour according to socially-derived inhibitory norms.

## Introduction

Attending to social cues and learning to interact with others are vital skills to develop in order to function successfully in society. Researchers have focused on the characteristics of individuals’ looking behaviour while engaged in social tasks (e.g. face recognition task). Social attention refers to the manner in which people attend to biologically relevant stimuli, in particular conspecifics [1]. Studying where people look has become one of the more widely-used methods for attempting to elucidate social attention [2]. As an indispensable tool for studying social attention, eye trackers provide a moment-to-moment record of where an individual is looking. Despite its widespread use and increasing sophistication, eyetracking is difficult to use covertly.

In the real world, people are aware of whether or not they are being observed and often modulate or alter their behaviour accordingly. Previous studies have shown that individuals are more likely to donate money [3], to offer help [4], or to cooperate [5] when they know they are being watched. Even exposure to eye-like images can increase cooperative or prosocial behaviour [6, 7, 8], reflecting one’s need to attain approval or avoid the disapproval of others. In social facilitation studies, it has also been reported that cues of being observed alter participants’ criteria for making decisions in non-social tasks (e.g., stroop task [9]; food intake [10]; visual search task [11]). These converging evidence clearly demonstrate how prosocial behaviour, decision making and task performance can be mediated by an increased self-awareness – a state that can be easily evoked by implicit observability cues. Considering these findings, researchers should not take for granted that people will behave normally when they know they are being watched, either in the real world or in lab-based experiments.

Interestingly, it does not require the physical presence of others to exert influence on behaviour [2, 12]. Previous research has shown that the presence of a camera can have various significant effects on human behaviour, from increasing pro-social behaviours [4] to altering cognitive performance [13]. When eye-tracking studies are conducted, participants usually know that they are being eye tracked. One of the basic assumptions behind an eye tracking approach is that natural looking behaviour is insensitive to the physical act of wearing an eye tracker and the knowledge that one’s eyes are being monitored. However, given that eye trackers are essentially video cameras for the eyes, tracking someone’s eyes could invoke mechanisms similar to those engaged by a more conventional video camera [4] or images of watching eyes [14]. Individuals may feel more self-conscious knowing their viewing behaviour is being directly monitored [10, 13]. Therefore, the use of the eye tracker might alter one’s natural viewing behaviour. Although most modern eye-tracking systems are now able to track participants’ eyes unobtrusively without using headgear and having their heads immobilized, the fact that a general lack of empirical data regarding the impact of mere belief of being eye-tracked on looking patterns exists suggests that it is worth some exploration.

In a closely related study, Risko and Kingstone [2] demonstrated that individuals are highly sensitive to their eye movements being tracked (i.e. the awareness of eye tracking). 24 undergraduate students were asked to complete an irrelevant computer-based task alone in an experimental room that contained a somewhat provocative stimulus (i.e. a sexy swimsuit calendar). Half of the participants knew their eyes were being monitored (via an eye tracker) and another half did not (via a hidden camera). Participants wearing the eye tracker were less likely to direct their gaze towards the ‘eye-catching’ calendar than those who performed the ‘natural looking’ task without an eye tracker. Hence, they concluded that wearing an eye tracker, an implied social presence, could alter individuals’ looking behaviour. They later repeated the study by including another condition in which participants wore the eye tracker but were informed that it was switched off. Remarkably, participants’ looking behaviour in the non-operational eye tracker condition resembled those not wearing an eye tracker. This demonstrated that the alterations in looking behaviour were likely due to the mere belief that one’s eyes are being tracked or not, instead of the physical act of wearing the eye tracker. These results also indicate that people will maintain prosocial looking behaviours and avoid acts that would damage their self-image when they are aware that their looking behaviour is being experimentally scrutinised. However, the study failed to test the possible influence of the content of stimuli on looking behaviour – for instance, the question of whether the implied social presence of an eye tracker affects the looking behaviour of individuals in ways other than viewing sexy calendars remains open to investigation.

Despite the increased attention to social observability cues [8,14-16], it remains unclear whether these behavioural findings derived from natural viewing tasks may generalise to eye-movement behaviour in laboratory-based tasks. The majority of eye-tracking studies of social attention have been conducted in a somewhat socially deprived manner, with a single participant sitting alone in an experimental room passively looking at biologically relevant stimuli, e.g., images of people. In contrast, in everyday life people exert top-down control over social attention in an active manner that is often divergent to what has been observed in the lab – for example, while studies conducted in laboratories demonstrated that people tend to predominantly direct their fixations to the eye region of a face presented in isolation [17, 18], studies conducted in real life situations found that people tend to avoid prolonged eye contact with targets [19, 20]. There is now converging empirical support that this disconnection is likely due to the absence of dual function of social gaze – communication and observation – when one is simply looking at static images of individuals (for a review, see [21]). That is, because static images of people neither observe one’s gaze nor communicate back, one’s own eyes merely serve to observe and do not communicate to the image [22]. Thus, in the lab it is perfectly acceptable to stare at the eyes of a stranger’s image, but in real life, prolonged eye contact can be perceived as a threat signal [23].

Indeed, how people attend to the social aspects of the world is influenced by the potential for social interactions [20, 24], social presence [1], and social norms [22, 25]. Although these critically relevant social information are often absent when examining eye movements in laboratory settings, it would be premature to conclude that social attention may never extend beyond the laboratory cubicle. Recent empirical research has clearly demonstrated that using more naturalistic stimuli and tasks involving potential social interactions could generate looking patterns that closely resemble those in natural social situations [26, 27, 28]. Unfortunately, studying social attention in relatively interactive contexts (i.e., face-to-face encounters) in real-time is not always feasible. Hence, it would be beneficial to see if the use of dynamic videos, coupled with the heightened awareness of being eye-tracked, would produce a more socially normative looking behaviour. Such laboratory-based work is particularly important because of its potential to enhance the ecological validity of eye-tracking studies on social attention without the necessity of conducting research under live/virtual social contexts.

## The Present Experiment

Eye-tracking studies have considered how people extract information from stimuli but have ignored one important signal – awareness of being eye-tracked. Accordingly, the present study was concerned with the possible impact of the awareness of being eye-tracked on individuals’ eye movement strategies in a computer-based task. We chose a modified task – a yes/no face recognition task – that we had good reason to believe it would involve active engagement with the dynamic stimuli. To assure that it is the knowledge that one’s eyes are being tracked and not the physical act of wearing the eye tracker accounting for the results, we use a remote, contact-free eye tracker to investigate if participants’ looking patterns were different when they knew they were being eye tracked compared to when they did not know. In order to conceal the main purpose of the present study, participants were told they would be participating in a simple face recognition study. To test the possible impact of the awareness of eye tracking, the face recognition paradigm here was coupled with the manipulation of being “watched” by the eye tracker. This manipulation was achieved by turning a dummy eye tracker ON (being eye tracked) or OFF (not being eye tracked) in front of the participants. Previous research indicates that being aware of the presence of eye tracker may trigger normative or socially desirable behaviour, arising from the need to present a positive image [2, 12]. We therefore expect that an awareness of eye tracking would induce changes in looking behaviour by implying the presence of an audience. If correct, people should demonstrate looking behaviour that is more in line with social norms when they are highly aware of being eye-tracked compared to when they are not.

In order to produce a more accurate and ecologically valid measure of eye movements, we utilised dynamic rather than static stimuli. While the stimuli were video recordings of people, and as such do not observe the participants, the knowledge that one’s eye movements are being tracked may function as an implied social presence by making informed participants believe that the eye movement recordings would later be examined by an experimenter. We speculated that presenting video clips, rather than static images, may generate stronger social observability cues, and thus activating norm-governing systems, guiding allocation of social attention.

The current study was designed to fill a gap in the literature by systematically investigating the extent to which the impact of eye-tracking awareness on eye movement pattern can be modulated by different types of stimuli used (social versus non-social stimuli). While staring at others’ eyes and bodies can be considered as rather impolite in social situations, such social rules do not typically apply to inanimate objects. The aforementioned rationale formed the basis of the present experiment that examined participants’ eye movement patterns in response to strictly controlled video clips displaying non-social (inanimate object) or social (face-only and face-with-body) stimuli. It was broadly hypothesized that participants’ eye movements would be unaffected by the knowledge of being eye-tracked while perceiving neutral inanimate objects, but could change substantially when the target is a face or face presented along with its associated body parts. More specifically, we anticipated that believing that one’s eyes are not being tracked (i.e. turning eye tracker off; misinformed condition) would yield looking behaviour that is deviant from the social norms in face-and-body (more fixations on the chest) and face stimuli (longer fixations on the eyes – considered disrespectful in East Asian cultures) [29]. Since there are typically stronger social taboos against staring at women than at men, particularly in Muslim cultures, it may be predicted that the implied social presence of the eye tracker may more strongly influence fixations on women’s than on men’s faces and bodies. Further, women have been found in previous studies to spend more time than men examining faces [30], possibly explaining their greater ability to identify nonverbally expressed emotions [31]. It may therefore be predicted that we may find women directing more fixations towards the faces than men.

## Methods

### Participants

66 Malaysian young adults attending the University of Nottingham Malaysia Campus participated in the study. Sample size was determined in advance based on two previous studies [2, 12] that found a strong implied social presence effect induced by an eye tracker, with group samples of 24 and 59 subjects, respectively. All participants self-reported normal or corrected-to-normal vision. Written informed consent was obtained from all participants and the protocol was approved by Faculty of Science Ethics Committee at the University of Nottingham. Data from 3 participants were excluded due to the issue of inaccurate calibration, excessive eye-tracking data loss (gaze samples under 50%), and procedural failure. Participants who were included in the analyses were 63 Malaysian young adults (32 males, 31 females; mean age = 19.62 years, *SD* = 1.54, age range: 18-25 years; 31 in the misinformed group and 32 in the informed group). All participants were non-psychology students who self-reported having very little to no experience with eye-tracking methodology (e.g., first time taking part in an eye-tracking experiment).

### Materials

Three main types of stimuli were used in the experiment: inanimate object, face-and-body, and face. To examine the impact of the awareness of eye tracking, short video clips with inanimate objects were used as the non-social stimuli while face only and face-and-body stimuli were used as the social stimuli. All videos were 1280 × 720 pixels in size and were muted.

Inanimate stimuli. 20 videos displaying inanimate objects (e.g. departing aeroplane, bouncing ball, swaying boat, etc.) were obtained from the Youtube website. The raw videos were cropped so that extraneous background details were excluded.

Face-and-body stimuli. Face-and-body stimuli were collected from 54 Malaysian young adult subjects (27 males, 27 females; age range: 18-24 years old). We refer to the individuals in the videos as “subjects”. Individuals taking part in the main experiment will be referred to as “participants”. The face-and-body stimuli were dynamic videos of “normal appearing” (no major facial lesions or deformities) students at the University of Nottingham Malaysia Campus. The experimenter who took the videos was well trained so that all the videos fulfil the same criteria: controlled studio lighting (non-flash), full head with upper body visible, frontal view, wearing a uniform grey shirt, and light grey background. Subjects were videotaped with all jewellery, makeup, and spectacles removed. While being video recorded, subjects were asked to look directly into the camera and to maintain a neutral, natural, and pleasant expression while verbally expressing a few sentences (i.e. introducing themselves briefly by saying their name, age, where they come from, what course they were studying and their hobby). All videos were recorded with a Panasonic HDC-TM300 digital video camera and subsequently cropped to include only the subjects’ head and upper body.

Face stimuli. The face stimuli were created by cropping the video clips obtained from the same 54 subjects so that only full head and shoulders were visible. All face videos were trimmed from different keyframes so that the facial expressions varied between study and recognition phase to avoid trivial matching strategies for memorizing faces.

### Apparatus

A Tobii T120 eye tracker was used to record participants’ eye movements. It uses infrared technology to measure corneal reflection in the observers without the use of a head-mounted device. The on-screen remote eye tracking system has an integrated infrared camera located beneath a 17” display monitor with a resolution of 1280 × 1024 pixels. The eye tracker performs binocular tracking at 120 Hz sampling rate by measuring the X and Y coordinates of the participants’ pupils while viewing the monitor and is accurate to within 0.4˚ visual angle. The minimum fixation duration and saccade thresholds were set to 100ms and 6 pixels/ms during the recordings. Tobii Studio software was used for stimulus presentation, data collection, and data coding. To manipulate participants’ awareness of eye tracking, two dummy webcams (Microsoft LifeCam VX-800) were placed at the top of the monitor and tilted slightly downward as if to record participants’ gaze. The original brand name on the webcams was concealed by official Tobii logo stickers to make participants believe that those were Tobii eye trackers. The webcams also feature LED lights that were rigged to allow them to be switched on and off by the experimenter to simulate the cameras being switched on and off.

### Procedure

Prior to the experiment, participants were asked to report demographic information, including age, gender, ethnicity, nationality, and course of study. Each of them was provided with a participant information sheet and a consent form so that they understood the tasks and agreed to take part in the study. They were then tested individually in a quiet room. Participants were asked to sit comfortably and approximately 60cm away from the monitor. Prior to testing, calibration was performed using a standard 9-point grid as implemented in the Tobii Studio software to ensure pinpoint accuracy. Participants followed a bouncing red dot with their eyes as it moved around the screen. The diameter of the red dot was changing from 0 to 2.5cm. If the participant’s fixation was more than 2.5cm (1˚) away from the centre of the dot, a re-calibration was performed until the optimal calibration criterion was achieved. After the system was well-calibrated, the learning block (Block 1) of the experimental phase began.

**Figure 1.**
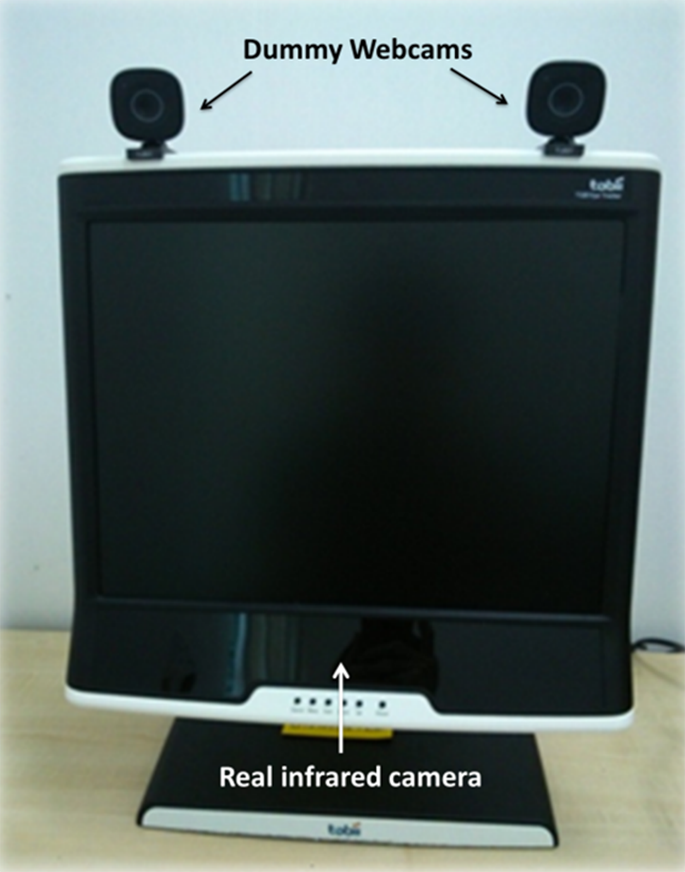
Equipment setup of the study, showing dummy webcams on top of the eye tracker screen.

Participants were randomly assigned to the informed or misinformed group. Prior to the experiment, both groups were told that the social images which they viewed during the learning phase had to be remembered for a later recognition task. To manipulate the level of eye-tracking awareness, participants were told that the webcams on top of the display monitor were “eye gaze cameras” (see Figure 1). They were led to believe that the “eye gaze cameras” were connected to the eye tracking system, which could record their eye movements. Yellow LED lights (power indicators) at the front of the “eye gaze cameras” could be turned on or off. Participants in the informed group were told that the “eye gaze cameras” were always on throughout the experiment. On the other hand, participants in the misinformed group were misled that a different group would be eye tracked, and that they were required to perform the calibration for the purpose of maintaining consistency between groups and only their behavioural responses would be recorded. After the calibration procedure, the power indicator lights were turned off for the misinformed participants, but left on for the informed participants. In fact, both groups were eye-tracked throughout the experiment. To ensure the potential changes in looking behaviour was not due to the physical presence of the experimenter, participants performed the tasks alone in the testing room after the calibration procedure.

**Figure 2.**
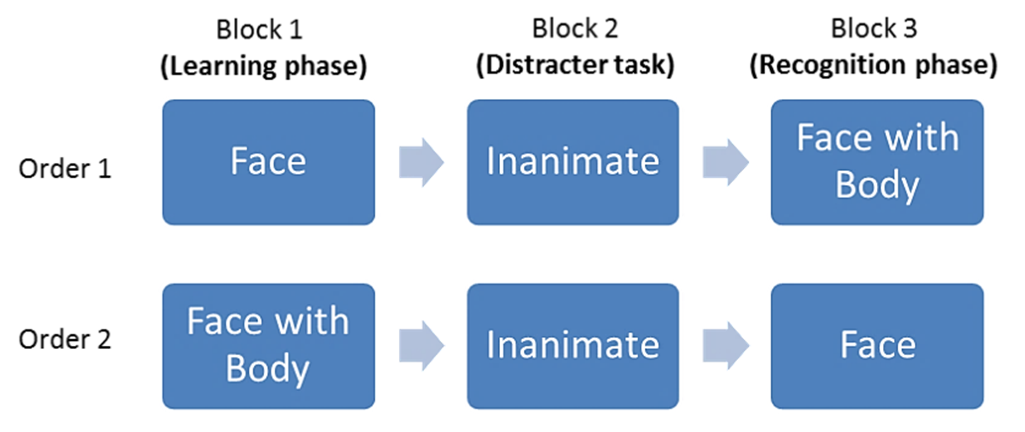
The orders of stimulus presentation in three separate blocks.

In fact, the face recognition task was used to conceal the true nature of the current investigation (i.e., awareness of eye-tracking). By employing a modified yes/no recognition paradigm, the experiment involved three phases: learning phase (Block 1), inanimate-object processing phase (Block 2), and recognition phase (Block 3). Block 1 and Block 3 contained either 36 face-and-body videos or 36 face-only videos, and the order of these two blocks was counterbalanced across participants. The inanimate stimuli were always presented in Block 2 (see Figure 2).

During the learning phase, 36 videos (18 male, 18 female) were shown one at a time and participants were asked to rate the faces for attractiveness on a seven-point Likert scale. Following the learning task, participants completed a 3-minute distracter task in which they viewed 20 videos of inanimate objects and then were required to rate the attractiveness of each video based on a seven-point scale. This was immediately followed by a recognition test in which another 36 videos (18 male, 18 female), of which half were targets and half were distractors, were presented. By means of a mouse click, participants were required to indicate if they had seen the person in each video before in the previous learning task.

In all experimental phases, each trial started with a fixation cross presented pseudo-randomly in one of the four quadrants of the screen for one second to avoid fixation bias, followed by a video presented in the centre of the screen. Each video was presented for 5 seconds and was followed by a question in relation to the task (e.g., “How attractive do you think this face it?” for the learning task and “Do you recognise this face?” for the recognition task).” Each response was subsequently followed by a fixation cross, which preceded the next video.

Following the experimental session, participants were asked the open-ended question “if you were asked to guess what hypothesis is being tested by this study, what would it be?” Twenty-one of the 32 participants in the informed group mentioned eye tracking or eye movements in their answers. None of the participants in the misinformed group reported eye tracking, eye movements or related phrases in their answers, indicating that they believed that the eye tracker was turned off and their eye movements were not being recorded. Participants in both groups guessed that the aim of the study was to investigate human face processing and recognition. Participants in the misinformed group were further asked whether they thought their eyes were being tracked during the experiment, and all replied that they did not. Therefore, the experimental manipulation – participants believing that they were or were not being eye tracked during the experiment – appears to have been effective.

Eventually, all participants were informed about the hidden (built-in) eye trackers at which point they had the option to consent or not to the use of that recorded eye tracking data. None of them withdrew consent. Finally, they were fully debriefed about the real purposes of the study, and the experimenter answered any questions regarding the meaning and procedure of the study.

### Area-of-Interest (AOI) Analysis

**Figure 3.**
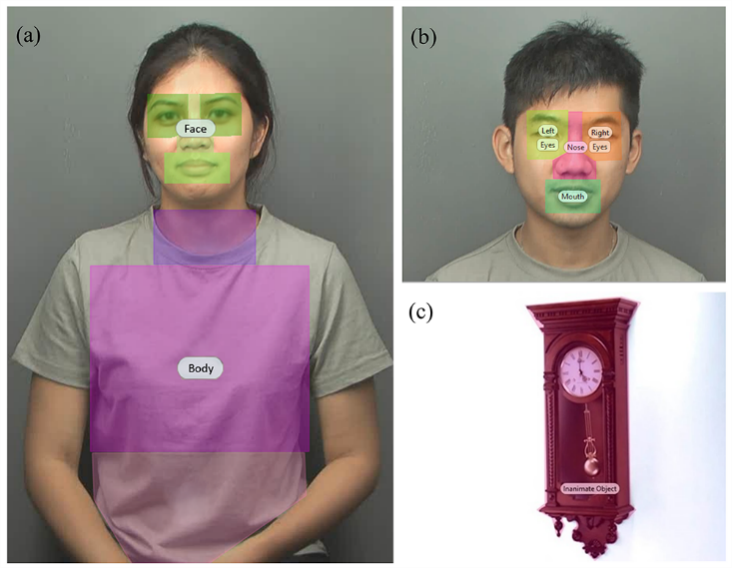
Examples of stimuli used with predefined areas of interest (AOIs): (a) face-and-body stimulus; (b) face stimulus; (c) inanimate stimulus.

For eye-tracking data analysis, AOIs were drawn for each target stimulus frame by frame in advance using Tobii Studio software so that the eye tracking system could capture and calculate the number of fixations and fixation time within each of these predefined AOIs. For inanimate stimuli, two main AOIs were drawn by separating the scene into inanimate objects and background. For each face stimulus, a general template of AOIs was created, outlining the nose, mouth, and eyes region (see Figure 3). Two AOIs were also created for face-and-body stimuli by drawing lines that divided the body parts in the videos into two main areas – face and body. In order to keep AOIs similar across face and face-and-body trials, the face AOI was made up of the nose, mouth and eyes regions from the face stimuli. The body AOI was made up of the neck, chest and waist. Only eye movements that fell within these predefined areas were analysed. During the experiment, AOIs were never visible to participants.

## Results

### Data Handling

Raw eye-tracking data were processed directly from the eye tracker using the Tobii Studio software. The area-of-interest (AOI) analysis was used to collect the sum of fixation counts and sum of fixation duration each participant made within the predefined areas per trial. In order to examine participants’ fixation patterns, we then computed the average number of fixations and average fixation duration per trial for each participant. All participants viewed 36 face-only, 36 face-and-body, and 20 inanimate videos, each presented for 5 seconds. Each trial began with the presentation of a fixation cross in one of the four quadrants of the computer screen, allowing the experimenter to check if the calibration was still accurate (drift correction < 1˚). To ensure that the temporally missing gaze data (e.g., due to inattention) were not included in the analyses, we removed a few trials in which there was zero fixation. Furthermore, only participants who had an average fixation count more than five counts across trials during each task were included in the data set. Three participants did not meet the criteria and were excluded from the eye tracking analysis.

### Behavioural Performance

A 2 (Awareness of Eye Tracking: Informed vs. Misinformed) × 2 (Gender of Participant: male or female) factorial analysis of variance (ANOVA) was conducted on participants’ face recognition accuracy (i.e. percent correct). No significant difference was found between informed ( *M* = 86.02%, *SD* = 6.75) and misinformed group (*M* = 84.74%, *SD* = 6.16), *F* (1, 59) = 0.634, *p* = .43, *η _p_^2^* = .011, showing that the awareness of being eye-tracked neither facilitated nor impaired subsequent task performance. Moreover, no gender difference was observed, *F* (1, 59) = 0.199, *p* = .66, *η _p_^2^* = .003.

Fixation Patterns: Inanimate Object

Average total fixation count. A 2 (Awareness of Eye Tracking: Informed vs. Misinformed) × 2 (Area of Interest: Inanimate object and Background) mixed factorial ANOVA showed a significant main effect of AOI, *F* (1, 61) = 1445.46, *p* < .001, *η _p_^2^* = .96, showing that participants fixated significantly more on the inanimate objects (*M*=10.74) than on the background scene (*M*=1.42). However, the main effect of awareness of eye tracking and the interaction between these two variables did not reach significance (*F* (1, 61) = 0.22, *p* = .65, *η _p_^2^* = .004 and *F* (1, 61) = 0.72, *p* = .40, *η _p_^2^* = 0.01, respectively.

Average total fixation duration. A similar mixed ANOVA conducted on participants’ average total duration of viewing time revealed a significant main effect of AOI, *F* (1, 61) = 1507.38, *p* < .001, *η _p_^2 ^*= .96, with longer fixation duration for the inanimate object (*M*=3.33) than the background ( *M*=0.32). However, the main effect of awareness of eye tracking and the interaction were found to be non-significant, ( *F* (1, 61) = 0.53, *p* = .47, *η _p_^2^* = .009 and *F* (1, 61) = 1.52, *p* = .22, *η _p_^2^* = .024, respectively), indicating that having the knowledge of being eye-tracked did not influence how participants would look at an inanimate stimulus. It is important to note that owing to the great physical variability within the set of inanimate stimuli, it was not possible to draw multiple AOIs with identical sizes on every inanimate object. Due to this limitation, we were only able to make detailed comparisons of fixation patterns for certain inanimate stimuli (Appendix A). One-way ANOVAs were performed separately on fixation number and fixation duration on each AOI, but again no significant difference between informed and misinformed groups was detected (see Table 1 in Appendix B for mean fixation data).

### Fixation Patterns: Face-and-Body Stimuli

Bonferroni-corrected paired samples t-tests did not reveal any significant impact of the task (learning vs. recognition) on the average total fixation count or duration for each AOI (all *p*s >.05). Therefore, data were collapsed across the two experimental phases in subsequent analyses. As the subjects of both genders were included in the face-and-body stimuli, we also wanted to check if the gender of stimuli interacted with gender of participant. For each measure, we conducted a 2 (Awareness of Eye Tracking: informed vs. misinformed) × 2 (Body Parts: Face and Body) × 2 (Gender of Participant) × 2 (Gender of Stimuli) mixed-factors analysis of variance (ANOVA).

Average total fixation count. A mixed factorial ANOVA revealed a significant main effect of Body Parts, *F* (1, 59) = 436.04, *p*<.001, *η _p_^2^* = .88. All participants showed more fixations on the facial regions more than on the bodies. However, neither a significant main effect of Awareness of Eye Tracking, *F* (1, 59) = 0.32, *p*=.57, *η _p_^2^* = .005, nor an interaction between Awareness of Eye Tracking and Body Parts was found, *F* (1, 59) = 1.77, *p*=.18, *η _p_^2^* = 0.03. There was a main effect of Gender of Participant, *F* (1, 59) = 5.47, *p*=.02, *η _p_^2 ^*= .09. Compared to male participants (*M*=4.30), female participants (*M*=5.03) made more fixations on the face-and-body stimuli presented. There was also a significant interaction between Gender of Stimuli and Gender of Participant, *F* (1, 59) = 5.82, *p* = .02, *η _p_^2^* = .09. Simple main effect analysis showed female participants fixated more at female ( *M* = 5.14) than male models (*M* = 4.91) ( *p* = .02) whereas there was no significant difference of number of fixations male participants made on stimuli of both genders ( *M_male_*=4.35; *M_female_*=4.26) (*p*=0.36). All other terms were not significant (all *p* >.05) and were not relevant to our main hypotheses.

**Figure 4.**
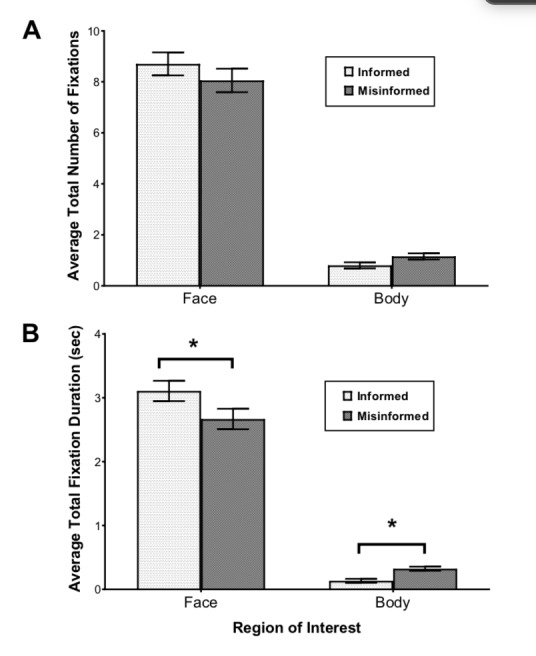
(A) Average total number of fixations and (B) average total fixation duration on faces and bodies as a function of awareness of eye tracking (informed vs. misinformed group). Error bars represent standard errors of the mean.

Average total fixation duration. A similar mixed factorial ANOVA on the average total fixation duration revealed a significant main effect of Body Parts, *F* (1, 59) = 459.83, *p*< .001, *η _p_^2^* = .89 and a significant interaction between Body Parts and Awareness of Eye-tracking, *F* (1, 59) = 4.39, *p*= .04, *η _p_^2^* = .07. Both the informed and the misinformed group fixated longer at the faces than at the bodies. The significant interaction between Awareness of Eye Tracking and Body Parts was primarily due to the fact that misinformed participants looked at the bodies significantly longer compared to informed participants ( *p*=.04) whereas informed participants fixated longer on the faces compared to misinformed participants (*p*=.05) (see *Figure 4*). However, the main effect of Awareness of Eye Tracking failed to reach significance, *F* (1, 59) =2.91, *p*= .10, *η _p_^2 ^*= .05. A main effect of Gender of Stimuli was found, *F* (1, 59) = 7.47, *p*=.008, *η _p_^2^* = .11, showing that female models (*M* = 1.56) received longer fixations than male models (*M* = 1.51).

The critical four-way interaction reached significance, *F* (1, 59) = 6.10, *p*=0.016, *η _p_^2^* = 0.09. To further understand what effects were driving this interaction, the data were split by participant group. For misinformed participants, the Gender of Stimuli × Gender of Participant × Body Parts interaction was highly significant, *F* (1,29) = 13.38, *p*=.001, *η _p_^2^* =.32. The significant interaction of Gender of Stimuli × Body Parts was evident in both male and female groups, *F* (1, 15) = 6.43, *p*=.02, *η_p_^2^* =.30, and *F* (1, 14) =7.73, *p*=.02, *η_p_^2^* =.36. Simple main effect analyses demonstrated that misinformed male participants looked longer at female faces than male faces (*p*=.01) while no significant difference was found for body fixations (*p*=.62). In contrast, misinformed female participants looked longer at female bodies than male bodies (*p*=.009) but no significant difference was found for face fixations (*p*=.24). For informed participants, however, the three-way interaction did not reach significance, *F* (1, 30) =.004, *p*=.95, *η_p_^2 ^*= .001, indicating similar eye movement patterns for own-gender and other-gender stimuli. All of the other main effects and interactions were not significant (all *p* >.05) and irrelevant to our hypotheses.

### Fixation Patterns: Face Stimuli

Average total fixation count. For face stimuli, 2 (Awareness of Eye Tracking: informed versus misinformed group) × 2 (Gender of Participant) × 2 (Gender of Stimuli) × 3 (AOI: eyes, nose, and mouth) ANOVAs were conducted separately on participants’ average total fixation count. Eye-tracking results did not show a significant main effect of Awareness of Eye Tracking, *F* (1, 59) = 0.42, *p* = .52, *η_p_^2^* = .007. The average number of fixations on each face region (i.e., eyes, nose, and mouth) did not differ between the informed and misinformed group, *F* (2, 118) = 0.10, *p*=.91, *η_p_^2^* = .002. A main effect of AOI was found, *F* (2, 118) = 5.73, *p* = .004, *η_p_^2^* = .09. Simple main effect analyses (with Bonferroni correction) showed that participants made significantly more fixations on the nose (*M*=5.07) than on the eyes (*M*=3.51) (*p* = .001) but no difference was found between the nose and mouth (*M*=4.55) (*p* =.76) and between the mouth and eyes (*p* = .20). A three-way interaction between Awareness of Eye Tracking, Gender of Stimuli and AOI was significant, *F* (2, 118) = 4.80, *p* = .01, *η_p_^2 ^*= .08. To further understand what effects were driving this three-way interaction we conducted a separate 2 (Gender of Stimuli: male, female) × 3 (AOI: eyes, nose, mouth) repeated-measures ANOVAs (Greenhouse-Geisser corrected) on each participant group, revealing that female faces produced a greater amount of mouth fixations (*p*=.02) but lower amount of eye fixations (*p*=.008) than male faces in the informed group. Yet, no significant difference was observed in the misinformed group (all *p* > 0.05).

**Figure 5.**
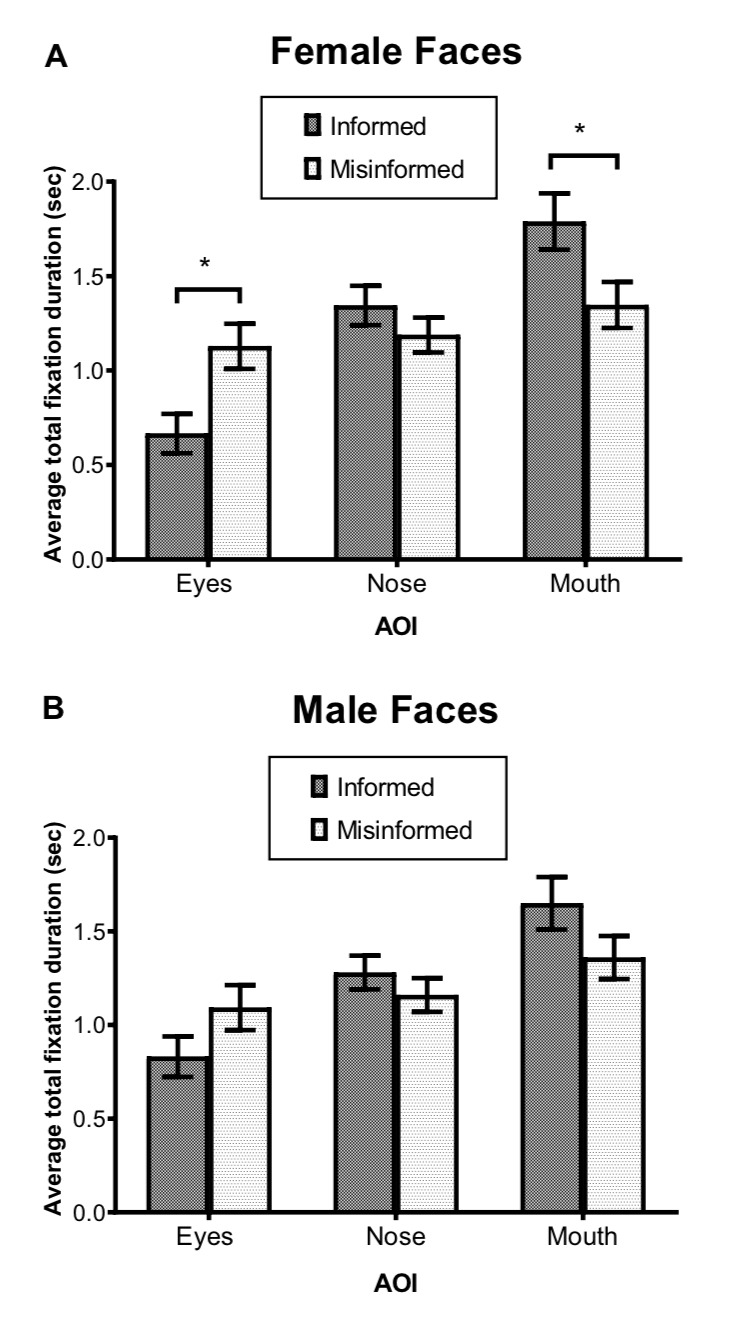
Average total fixation duration as a function of the areas of interest (AOI) for informed and misinformed groups for (A) female faces and (B) male faces. Error bars show standard errors of the mean.

Average total fixation duration. A 2 (Awareness of Eye Tracking: informed versus misinformed group) × 2 (Gender of Participant) × 2 (Gender of Stimuli) × 3 (AOI: eyes, nose, and mouth) ANOVAs were conducted separately on participants’ average total fixation duration. Eye-tracking results did not show a significant main effect of Awareness of Eye Tracking, *F* (1, 59) = 1.53, *p* =.22, *η_p_^2^* =.03. A main effect of AOI was found, *F* (1, 118) = 11.40, *p* < .001, *η_p_^2^* = .16. Simple main effect analyses (with Bonferroni correction) showed that participants fixated longer at the mouth (*M*=1.57) than the nose (*M*=1.20) (*p* = .04) and eyes (*M*=0.87) (*p* < .001), and the nose longer than the eyes (*p* = .007). Most importantly, there was a significant interaction between AOI and Awareness of Eye Tracking, *F* (2, 118) = 3.96, *p*=.02, *η_p_^2 ^*= .07, indicating that misinformed participants made longer fixations on the eye regions (*p*=.02) whereas informed participants had longer fixations on the mouth (*p*=.05). A closer examination on fixation duration data revealed a significant three-way interaction between Awareness of Eye Tracking, Gender of Stimuli, and AOI, *F* (2, 118) = 5.45, *p* = .005, *η_p_^2 ^*= 0.09. Informed participants fixated longer on the mouth but shorter on the eyes of female faces than misinformed participants (mouth: *p* = .03; eyes: *p* = .005). Yet, no significant interaction was observed for male faces (all *p* > 0.05) (see *Figure 5*).

## Discussion

Although previous studies have demonstrated the psychological effect of being watched by others as an effective tool in altering social behaviour, the present experiment sought to investigate whether the awareness of eye-tracking could influence individuals’ eye movement behaviour in a laboratory setting. The current results support the notion that eye trackers may inhibit norm-violating looking behaviour but encourage more socially-acceptable looking patterns.

Behavioural results showed that the manipulation of being “watched” by the eye trackers did not affect participants’ recognition performance. Informed participants who were aware of their eyes being monitored performed no better in the yes/no recognition task than those who were not. As predicted, eye-tracking results did not reveal any significant difference in average viewing time and fixation counts between the informed and misinformed groups when presented with videos of inanimate objects (non-social stimuli), indicating that having the awareness of eye tracking did not influence how participants would look at a neutral inanimate stimulus.

For face stimuli, however, informed participants spent more time looking at the mouth but less on the eyes than misinformed participants, suggesting that they were more likely to conform to the East Asian social norm of avoiding prolonged eye contact when they believed they were being eye tracked. Such eye contact norms are commonly observed in many Asian societies where individuals are socialised to avoid sustained eye contact during face-to-face encounters [29, 32]. Another potential explanation is that participants might find dynamic movement of the mouth “eye-catching” and therefore distribute more attention to it in order to gain visual speech information from the muted videos of actors briefly introducing themselves. A less intuitive explanation may be that the lower half of a face provides an important source of diagnostic information which is advantageous for face recognition when the faces are dynamically displayed. The latter suggestion is in line with existing studies which have shown that the mouth region may comprise crucial cues for emotional facial expressions [33], and that East Asians’ nose-centric fixation pattern might facilitate holistic processing for faces in general [34, 35, 36].

For face-and-body stimuli, participants fixated more and longer at faces than at bodies, showing that people tend to gather information about others through viewing facial characteristics or expressions. Importantly, informed participants (who knew their eye movements were being recorded) fixated more and longer on the faces than misinformed participants, but were less likely to stare at the body regions (i.e. neck, chest, waist) than misinformed participants. This may reflect the fact that fixating on others’ bodies is a violation of a social norm, and thus participants avoided doing so when aware that their gaze was being monitored.

By focusing on the effect of eye-tracking awareness on looking behaviour, it appears that the attentional mechanisms responsible for eye-tracking awareness in a natural viewing task [2] and natural searching task [12] may also apply to a computer-based task in a laboratory. In line with Risko and Kingstone [2], our results suggest that awareness of being eye tracked can serve as an implied social presence that induces individuals to modify their natural looking behaviour to maintain a positive image in the context of being observed. Misinformed participants, who lacked that implied social presence, appeared less bound by social norms of gaze direction.

In most societies, especially in a more conservative East Asian country, staring at others’ bodies regions can be considered inappropriate. In order to maintain positive impressions, informed participants with increased self-awareness might resist the temptation to look at the bodies of models when they knew that the recorded eye movements may later scrutinised by an experimenter. Although it is impossible for the subjects in the video stimuli to look back at the participants, the sense of their gaze being recorded alone may activate the norm-governing system, and thus influence looking behaviour. In other words, the mere belief of being eye-tracked modulates participants’ top-down control over social attention.

Previous findings indicated that when viewing clothed models, participants tended to fixate more often on the facial rather than body region of the photographs [37]. In the current study, participants were presented with videos of clothed models (wearing plain grey t-shirts) where the outline of body shape and breasts (for female models) was clearly visible. These stimuli are not as provocative as the calendar with sexy models wearing swimsuits used in the study by Risko and Kingstone [2]. The differences in stimuli used could explain why the impact of awareness of eye tracking on participants’ looking behaviour was less pronounced in the current study. It may be predicted that if the models were depicted in a more provocative manner, a more prominent effect of eye-tracking awareness would emerge.

Although the awareness of being eye-tracked did not affect participants’ face recognition performance, it exerted a significant effect, compelling participants to direct their overt attention (as indicated by fixations) on socially salient stimuli congruently with established social norms, even in a laboratory context. This finding has potentially important implications for cognitive scientists who attempt to use eye-tracking technology to uncover the mechanisms underlying human perception, attention and memory with social stimuli. It raises important issues about the use of eye trackers in socially-salient, person perception tasks if eye trackers often fail to capture natural eye movements in such circumstances.

However, the current findings also offer opportunities to improve current eye tracking methodology. For example, if one wished to deter the eye-tracked observers from looking at things they normally would not look at in social contexts, he or she could place reminders into the recording software to keep observers aware that their gaze has a witness. Our findings also have practical implications for marketers who aim to measure customers’ visual engagement using eye tracking methodology in advertising research. It makes intuitive sense to examine the usability and effectiveness of ad designs by recording what people look at (or don’t look at) on a webpage, but given the susceptibility of eye movements to the influence of eye-tracking awareness, caution is needed in interpreting the data, especially when socially salient or provocative stimuli (e.g., sexy female models wearing bikini, muscular male models) are involved. In this case, one should try to avoid cues that remind participants of the fact that their eyes are being tracked.

In our study, the dummy eye trackers were clearly visible; it was also pointed out to the participants in the informed group that their eye movements were being monitored throughout the experiment, and the calibration procedure might also serve as a reminder that triggers their eye-tracking awareness [12]. These procedures not only made them more aware of the eye tracker but also led to the belief that the dummy eye trackers were actively monitoring their gaze behaviour. Nasiopoulos et al. [12] investigated whether the implied social presence effect triggered by an eye tracker is a transient or a sustained strong effect. They found that the prosocial effect of an eye tracker can be abolished in less than 10 minutes of wearing it, suggesting that eye trackers induce a transient social presence effect which becomes less pronounced over time. However, the authors also discovered that the implied presence effect can be easily reactivated by drawing attention back to the eye tracker. Although this was not directly tested in our computer-based experiment which took approximately 30 minutes to complete, the observed changes of fixation patterns caused by the increased eye-tracking awareness (triggered by the reminders during calibration procedure and experimental instructions) in the informed participants fit well with their latter finding. Additionally, no difference in fixation patterns were found between the learning and recognition phases of the study, suggesting that the influence of the implied social presence of the eye tracker was maintained across the duration of the study.

Although this study was not primarily aimed at investigating the gender differences in looking behaviour, some of the results bear on this aspect. Interestingly, female participants looked more at own-gender models than opposite-sex models in the face-and-body video stimuli, suggesting social comparison motivation [37]. They also spent a greater amount of fixations and viewing time attending to a model’s face compared to male participants. A recent study by Heisz et al. [30] suggested that women can recognize faces better than men because they spend more time studying facial features. Yet, our analysis did not reveal gender differences in face recognition performance. Besides, it has been reported that women have higher capability in decoding nonverbal emotion by looking more at the main parts of the face compared to men, with greater reliance on the eyes [31]. Therefore, one possible explanation is that female participants paid more attention to the faces to evaluate the emotionally neutral faces.

On the other hand, male participants tended to look more and longer at the models’ bodies than female participants did. Male participants’ visual attention to male models’ bodies could be interpreted a common tendency of men comparing own body shape with same-sex counterparts [38]. From an evolutionary perspective, men’s attention is more drawn to the sexual parts of the female body, especially the chest and waist, as they are thought to signal mate quality and reproductive potential [39, 40, 41]. Taken together, our results suggest that sexual body parts are attended to differently for male and female bodies by both male and female perceivers.

Until present, the majority of studies on social attention made use of experimental designs in which static stimuli were presented to participants. While these studies provided important insights into the underlying mechanisms of social attention, they may fail to capture an essential aspect of real-life social encounters. Previous research has found that gaze behaviour to faces depends on degrees to which stimuli are both social and dynamic [42, 43]. This concurs with neuroimaging evidence showing that the face-selective cortical region responds more strongly to dynamic faces as compared to static faces [44]. One strength of the present study is the use of dynamic stimuli, which are more realistic as compared to static images, and may therefore amplify the eye-tracking awareness effect. Perhaps the use of dynamic social stimuli (with direct gaze from the subjects) paired with a heightened awareness of being eye-tracked would produce more socially normative looking patterns. Future studies could consider exploring whether less naturalistic looking stimuli (e.g., static images) can modulate the effect of eye-tracker awareness.

It is tempting to interpret our results as showing that eye-tracking awareness exerts its influence only on social stimuli more strongly than on non-social stimuli. However, due to the differing numbers and categories of AOIs between the three different stimulus types, it is not possible to make this comparison directly, and so caution should be exercised in drawing this conclusion.

In sum, the current study demonstrated that an awareness of being eye-tracked systematically affects looking patterns in socially-salient stimuli. We argue that the eye tracker implies the presence of an audience, watching the eye movements made by participants, suggesting that the process whereby an implied presence affects looking behaviour involves heightened awareness of eye-tracking. Our additional analyses of the modulatory effect of participants’ gender also provide meaningful insights into the gender differences in social attention to face and body regions. If someone knows their eye movements are being monitored, they tend to demonstrate social-norm-based looking behaviour. The general idea that an eye tracker can play a role as an implied social presence is noteworthy, particularly for eye-tracking researchers investigating social attention in both laboratory and real-world settings. The current work not only bolsters a deeper understanding of social attention as well as implied social presence effects, but also indicates the necessity to re-evaluate the ecological validity of previous laboratory work on social attention using eye-tracking. Future research is certainly needed to understand the mechanisms underlying how such effect of eye-tracking awareness acts on looking behaviour.

## Ethics and Conflict of Interest

The authors declare that the contents of the article are in agreement with the ethics described in http://biblio.unibe.ch/portale/elibrary/BOP/jemr/ethics.html and that there is no conflict of interest regarding the publication of this paper.

## Acknowledgements

The authors are grateful to all the participants who took part in this experiment.

## Figures and Tables

**Appendix A:**
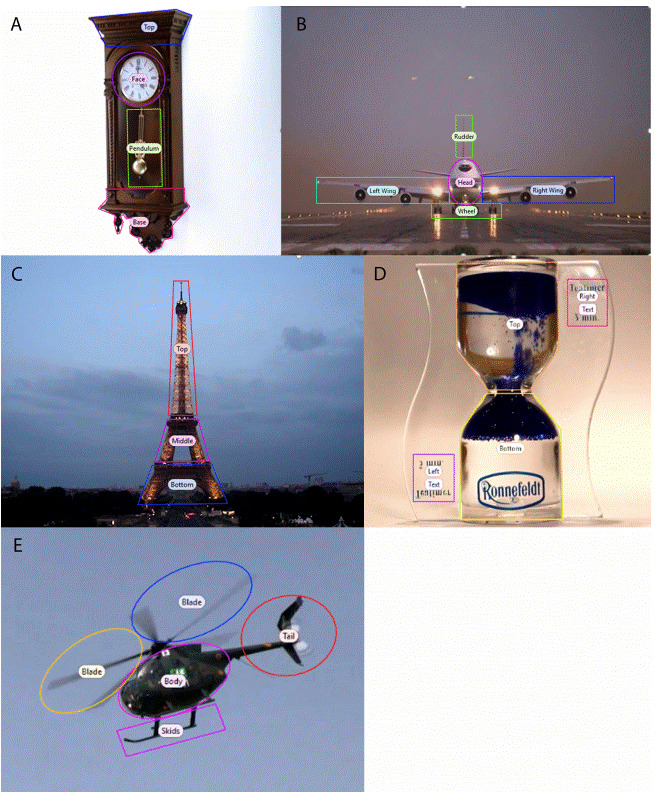
Specific AOIs predefined on particular inanimate stimuli used in the experiment: (a) clock, (b) plane (c) the Eiffel Tower, (d) hourglass and (e) helicopter.

**Appendix B:**
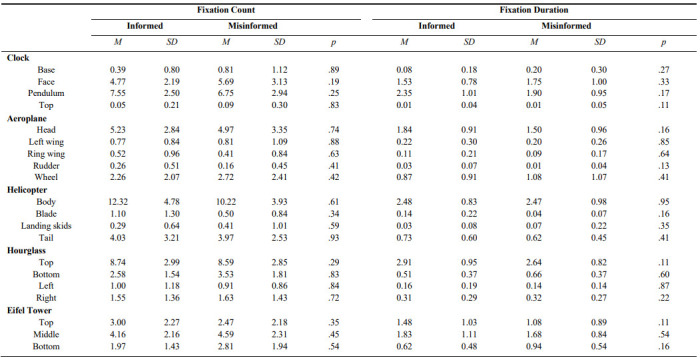
Raw number of microsaccade-response pairings included in each condition. Data was averaged for each participant over the 24 trials, prior to the following analysis.
